# The aesthetic experience of general beauty and ugly-cute memes: the role of emotion

**DOI:** 10.3389/fpsyg.2024.1340552

**Published:** 2024-04-25

**Authors:** Juan Li, Yi An, Tiansheng Xia

**Affiliations:** ^1^School of Educational Science, Hunan Normal University, Changsha, China; ^2^School of Art and Design, Guangdong University of Technology, Guangzhou, China

**Keywords:** general beauty, ugly-cute, emotion, humor, surprise, aesthetic

## Abstract

Generally, beauty has been regarded as an outward expression of elegance and harmony, providing visual pleasure and evoking a sense of aesthetic enjoyment. However, in recent years, a phenomenon called “ugly-cute” has emerged, challenging the conventional standards of beauty by embracing a form of “ugliness” to enhance its appeal. The reasons and mechanisms behind this phenomenon remain largely unexplored so far. This study aims to investigate the role of emotions, such as pleasure, humor, and surprise, in the relationship between ugly-cute characteristics and attractiveness. The findings reveal that general beauty directly generates attractiveness by eliciting pleasurable emotions, whereas ugly-cute memes achieve attractiveness by inducing pleasurable emotions through the mediation of humor. Furthermore, while both “ugly” and “ugly-cute” memes evoke a sense of surprise, that elicited by ugly-cute memes is accompanied by a humorous response, thereby enhancing their attractiveness, whereas the “ugly” memes fail to evoke humor and lack attractiveness. Finally, we discuss the potential implications and practical value of the current research.

## 1 Introduction

Attractive aesthetic objects are typically considered visually pleasing, and there is a general consensus among people regarding the characteristics of beauty (Carbon, 2011; Brielmann et al., 2021) such as fluency, symmetry, typicality, and complexity, among others. However, many highly attractive objects do not conform to these feature standards and may even be considered unattractive from a general beauty perspective. This phenomenon is particularly prevalent among Internet memes. For example, Lenda et al. (2020) generated interest in the less attractive *Nasalis larvatus* by presenting it in the form of Internet memes, significantly enhancing its attractiveness and serving as a means of promoting the conservation of endangered animals. The globally popular “Ugly Dolls” series of plush toys and movies also stand out due to their ugly-cute features, and they have gained immense popularity through Internet memes (Sabina, 2019). Similarly, in the Chinese movie “Ne Zha” (Fan, 2021), the character Ne Zha represents this “ugly-cute” aesthetic. Ne Zha’s “smoky makeup” and “buck teeth,” which are traditionally considered unattractive features, contrast with his high attractiveness, creating an intriguing aesthetic phenomenon.

Internet memes are “popular culture units that are spread, imitated, and transformed by individual internet users, creating a shared cultural experience” (Shifman, 2013). The internet has long-term aesthetic trends, one of which is “Internet Ugliness,” a previously unnamed style pervasive across many different internet cultures, particularly in meme content (Douglas, 2014). For instance, images with captions based on cultural elements can be termed as memes. Usually, memes convey a social message through a humorous format (Akram et al., 2020). We collectively refer to this category of memes, which have low aesthetic appeal but high attractiveness, as “ugly-cute.” As the name suggests, “ugly-cute” combines both “ugly” and “cute” elements. While the study of “ugliness” as an extreme expression of beauty has a long history in aesthetics and has been thought-provoking (Bayley, 2012), the experience of ugliness also has an emotional dimension. Artworks can evoke various emotions, varying in degrees of value and arousal (Leder et al., 2004; Markovic, 2010). However, most empirical aesthetic studies focus on the mixture of pleasure and displeasure aesthetic experiences, neglecting the more complex forms of aesthetic engagement associated with ugliness (Brady and Prior, 2020).

Another element of “ugly-cute” memes is “cute.” In recent years, there has been an increasing interest in the study of “cuteness” worldwide (Lieber-Milo and Nittono, 2019; Nittono et al., 2023). A survey conducted in Japan, the United States, and Israel shows that “cuteness” is associated with positive emotional responses (Nittono et al., 2021). Research on ugly-cute memes is beneficial for expanding the scope of aesthetics, challenging general definitions of beauty, and providing design strategies to enhance the attractiveness of objects with low aesthetic appeal. This study aims to explore the mechanisms through which emotions contribute to the high attractiveness of ugly-cute memes. As many have observed, the ugly-cute concept in visual art is captivating, emotionally moving, and often closely related to beauty, but it requires its own unique investigation.

Aesthetic experience is *a priori*, a unique, and even sublime form of human experience (Bara et al., 2022). Leder et al. (2013) investigated the factors influencing aesthetic experience in art portraits, and the study found that attractiveness (likability) notably impacts aesthetic preferences. However, although attractiveness is a significant aesthetic factor, it is not always directly related to the overall aesthetic appeal or quality evaluation of a product (Hoegg et al., 2010). The complete aesthetic response involves perceptual processing, cognitive evaluation, cultural knowledge, and emotions such as pleasure or surprise (Leder et al., 2004; Marković, 2012). “Aesthetic emotion” refers to the emotions evoked when appreciating aesthetic objects (Perlovsky, 2014; Silvia et al., 2015). Aesthetic objects possess unique stimulus features that can elicit specific emotions or emotional responses in observers (Menninghaus et al., 2019). Emotions play a significant cognitive role and have an important influence on aesthetic judgment (Tiihonen et al., 2024). Individuals experience pleasurable emotions when appreciating nature, artworks, and other human creations (Armstrong and Detweiler-Bedell, 2008). Researchers define “aesthetic pleasure” as a shared enjoyment derived from responding to the purpose or meaning expressed by an aesthetic object (Reber et al., 2004). The Pleasure-Interest Model of Aesthetic Liking (PIA model) developed by Graf and Landwehr (2017) suggests that aesthetically pleasing things elicit positive emotions such as pleasure and interest, thereby generating attractiveness. A recent review of studies suggests that, in addition to the aesthetic pleasure and interest emphasized in the PIA model’s automatic and controlled processing stages, aesthetic immersion pleasure is also present in the integrative sublimation stage (Menninghaus et al., 2019).

Therefore, our first hypothesis is:

*H1a*: Beauty positively influences pleasure.

*H1b*: Beauty positively influences attractiveness.

*H1c*: Pleasure positively influences attractiveness, acting as a mediator between beauty and attractiveness.

However, the attractiveness of ugly-cute memes may follow different pathways compared to general beauty. In the context of ugly-cute memes, this aesthetic immersion pleasure in the integrative sublimation stage may be influenced by other factors such as the emotion of humor. Humor, as a positive emotion, also plays a significant role in aesthetic experiences (Eisend, 2009; Gordon, 2012, 2013). Research has shown that humor can enhance positive emotional responses. For example, Cancelas-Ouviña (2021) found that memes with humorous features helped alleviate feelings of sadness, fear, and pain. In recent years, numerous studies have demonstrated the benefits of humorous memes for the psychological well-being of individuals with depression (Akram et al., 2020; Akram and Drabble, 2022). According to the incongruity resolution theory (Suls, 1972), individuals experiencing humor in a story first encounter a stage of incongruity, which precedes its resolution. The cognitive process underpinning humor comprehension consists of identifying and resolving the incongruity. Appreciating humor involves a deeper exploration of this comprehension, ultimately leading to the experience of enjoyment (Chang et al., 2024). Therefore, we hypothesize that ugly-cute features may elicit an emotional experience of humor, resulting in pleasurable emotions and subsequently increasing the attractiveness of ugly-cute memes.

Hypothesis two is as follows:

*H2a*: Ugly-cute positively influences humor.

*H2b*: Humor positively influences pleasure, which in turn positively influences attractiveness, suggesting that humor and pleasure mediate the relationship between ugly-cute and attractiveness.

Philosophical theories of beauty have detailed the contributions of various experiential dimensions to aesthetic experience, one of which is surprise (Brielmann et al., 2021). Brielmann et al. (2021) propose that a good aesthetic experience has six dimensions: intense pleasure, universal appeal, desire for continued experience, exceeding expectations, perceived diversity, and meaning. The design of ugly-cute memes often evokes surprise, which can be understood as “exceeding expectations.” Declos (2014) suggests that surprise can be regarded as a significant emotional response in aesthetic experiences, creating cognitive shifts and new understandings, thereby enhancing the appeal of artworks. According to Marmur and Koichu (2016), surprise plays a significant role in the aesthetic experience. Their research explored the aesthetic experiences of university students with mathematical problems, particularly the feelings of surprise elicited upon discovering unexpected solutions. The study found that the students’ aesthetic responses to the problems were largely dependent on the degree of surprise they experienced after revealing a clever solution, especially following several failed attempts. This suggests that surprise can enhance the aesthetic experience, making it more profound and memorable. Therefore, we hypothesize that surprise is also an important influencing factor in ugly-cute memes.

Hypothesis three is as follows:

*H3a*: Ugly-cute positively influences surprise.

*H3b*: Surprise positively influences pleasure, which subsequently positively influences attractiveness, indicating that surprise and pleasure mediate the relationship between ugly-cute and attractiveness.

## 2 Materials and methods

A total of 214 undergraduate and graduate students from Guangdong University of Technology participated in this study. Among the 214 participants recruited, 30 volunteers participated in the pre-experiment. Based on these assessments, our stimulus materials for formal experiment were determined. The remaining 184 participants engaged in the questionnaire assessment of emotions and attractiveness associated with the images. The study was conducted in accordance with the Declaration of Helsinki and approved by the Ethics Review Committee of the School of Guangdong University of Technology (Approval No. GDUTXS2023193).

### 2.1 Preliminary experiment

The appearance of ugly-cute products can include various memes, such as animals, mythical creatures, and cartoon characters. This study adopted a comprehensive approach that involved multiple categories. We obtained memes related to the theme through Internet searches using the keyword “ugly-cute,” and retrieved a total of 79,400 ugly images, of which we screened 204 for high viewing and more popular images. All the memes had a resolution of 300 dpi, and their size was standardized to 10 × 10 cm.

**Figure 1:**
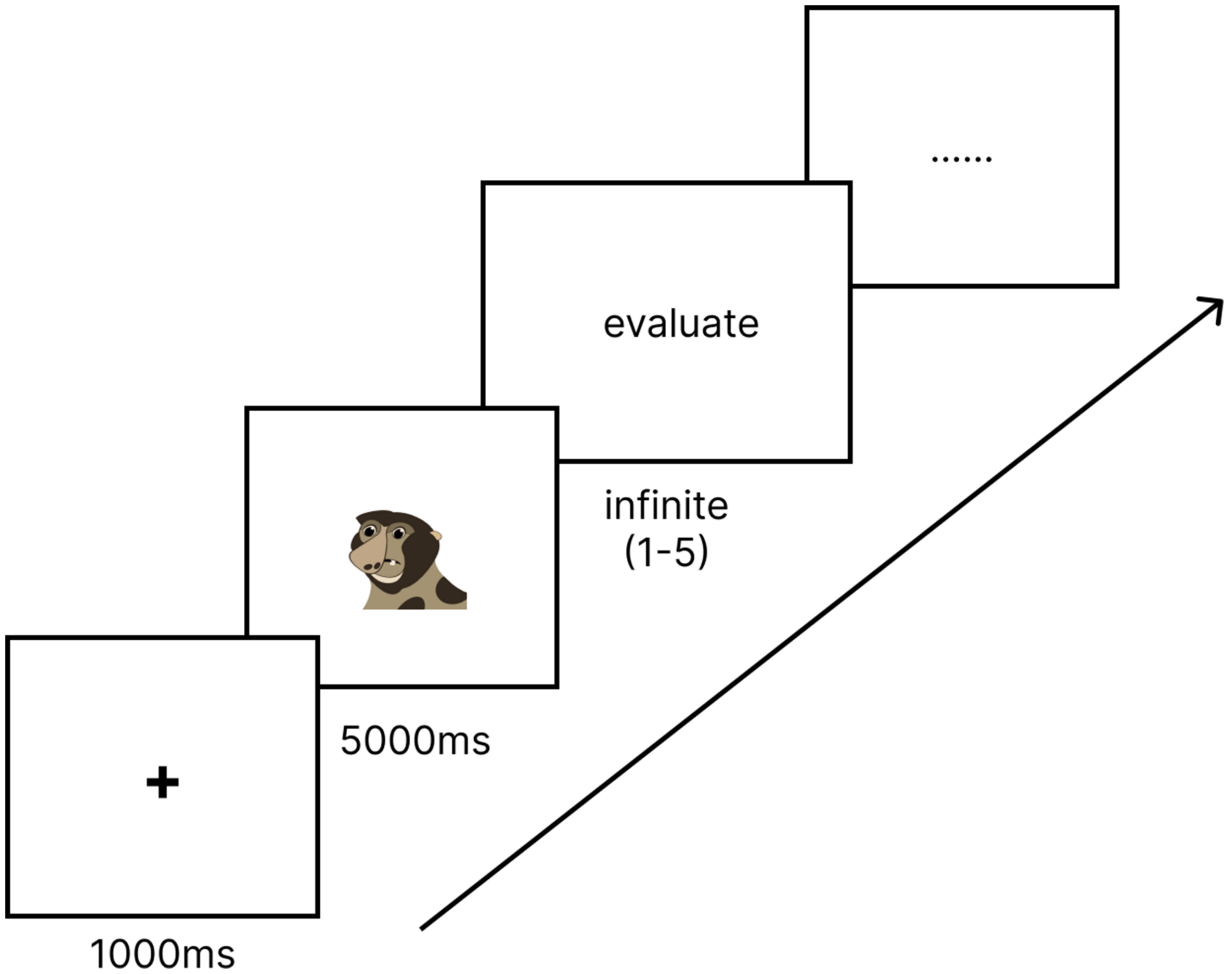
Screening procedures for stimulus materials. Image drawn by the authors.

Subsequently, we invited 30 volunteers (20 undergraduate and 10 graduate students) to evaluate the materials. Among them were 13 males and 17 females, aged 18 to 29 years (*M* = 21.80, *SD* = 3.03). These volunteers were recruited offline to evaluate the experimental materials. To control for the duration of image viewing, we used the E-Prime software (version 3.0) to create an experimental program for stimulus material selection (see Figure 1). The program comprised two parts, each containing 204 stimulus memes. These memes were randomly presented at the center of the screen, with each stimulus meme measuring 10 × 10 cm.

Before the experiment began, participants were required to fully understand the instructions and enter the practice phase. At the start of each trial, a fixation point “+” was displayed in the center of the screen for 1,000 ms. Subsequently, a stimulus image randomly appeared in the center of the screen and remained visible for 5,000 ms. During the presentation of the memes, participants were asked to rate their level of cuteness and beautiful. In the first part, participants were asked to answer the question: “Do you think this image is ‘cute’?” In the second part, participants were asked, “Do you think this image is beautiful?” Participants could rate their responses by directly selecting the corresponding keys, with a rating scale ranging from 1 (strongly disagree) to 5 (strongly agree). A total of 408 trials were conducted in the experiment.

### 2.2 Results of stimulus material selection

Based on participants’ ratings of the general beauty and cuteness levels of the stimuli, we categorized general beauty level and cuteness level into three: high, medium, and low. For general beauty, the top 1/3 of the ratings (more than 66% of the ratings, >3.58) were classified as high general beauty, while the bottom 1/3 of the ratings (less than 33% of the ratings, <3.07) were classified as low general beauty. For the cuteness level, the top 1/3 of the ratings (more than 66% of the ratings, >7.76) were classified as high cuteness, while the bottom 1/3 of the ratings (less than 33% of the ratings, <3.34) were classified as low cuteness. We divided the stimulus memes into three groups: low cuteness and high general beauty memes (beautiful group), low cuteness and low general beauty memes (ugly group, with a low level of general beauty and cuteness), and high cuteness and low general beautiful memes (ugly-cute group), with each group containing six memes. We then evaluated aesthetic perception, emotions, and attractiveness based on the selected memes in the formal experiment.

### 2.3 Formal experiment

#### 2.3.1 Participants

A total of 184 participants participated in the formal experiment. All participants were recruited through the online platform Sojump, which is a widely used online survey and questionnaire creation platform in China. Among them were 75 males and 109 females, ranging in age from 18 to 40 years (*M* = 22.27, *SD* = 3.71). Of the participants in formal experiment, 96 were college students and 88 were graduate students. All participants provided informed consent and received CNY 10 compensation for their participation.

#### 2.3.2 Design and stimuli

This study employed a 2 (beautiful degree: beautiful vs. ugly) × 2 (cuteness: cute vs. uncute) within-subjects design. Considering the primary focus of the current study is on the aesthetic mechanism of the “ugly-cute” phenomenon, we selected stimuli only at three levels: beautiful-uncute (beautiful group), ugly-cute, and ugly-uncute (ugly group). The current study manipulates the attributes of these stimuli as independent variables, while recording the participants’ emotional responses (pleasure, humor, and surprise) and aesthetic judgments (perceived attractiveness).

A total of 184 individuals participated in the formal experiment, and 181 valid questionnaires were collected. The evaluation was based on the measurement approach used by Schindler et al. (2017) for aesthetic emotions, which included six dimensions: beauty, cuteness, humor, surprise, pleasure, and attractiveness (see Table 1). The six dimensions were carefully selected based on their relevance to the evaluation of aesthetics, emotion, and attractiveness. Each item represents a specific aspect that contributes to the overall perception of beauty and appeal. Participants are asked to rate each item on a scale, indicating their level of agreement or disagreement with the statement. A five-point Likert scale was used, and participants were required to answer the following six questions (1 = strongly disagree, 5 = strongly agree).

**Table 1:** Items for the evaluation of aesthetics, emotion, and attractiveness.

Item	Answer
Beauty	It is beautiful and not ugly at all.
Cuteness	To me, it appears cute and adorable.
Humor	I was amused; it was funny to me.
Surprise	I was amazed and astonished by it.
Pleasure	It delighted me and made me happy.
Attractiveness	I was attracted and enraptured by it.

## 3 Results

### 3.1 General beauty and attractiveness

#### 3.1.1 Differential analysis general beauty and ugly

First, the means and standard deviations under each condition were calculated (see Table 2). To compare differences between general perceptions of beauty and ugliness, we conducted a repeated measure analysis of variance to evaluate variations in perceived beauty, emotional responses, and attractiveness under two conditions. Results showed significant differences in aesthetics between the beautiful group (*M* = 4.28, *SD* = 0.68) and the ugly group (*M* = 1.80, *SD* = 0.90), *F*(1, 180) = 952.07, *p* < 0.001, *ŋ_p_^2^* = 0.841. For perceived attractiveness, there were also significant differences, with the beautiful group (*M* = 4.02, *SD* = 0.83) and the ugly group (*M* = 1.79, *SD* = 0.87), *F*(1, 180) = 596.19, *p* < 0.001, *ŋ_p_^2^* = 0.768. In terms of pleasure, the beautiful group (*M* = 4.14, *SD* = 0.80) was significantly higher than the ugly group (*M* = 1.80, *SD* = 0.83), *F*(1, 180) = 689.70, *p* < 0.001, *ŋ_p2_* = 0.793. In humor, the beautiful group (*M* = 2.89, *SD* = 1.05) was significantly different from the ugly group (*M* = 1.99, *SD* = 0.87), *F*(1, 180) = 103.41, *p* < 0.001, *ŋ_p_^2^* = 0.365. However, for surprise, the beautiful group (*M* = 2.26, *SD* = 1.01) was significantly lower than the ugly group (*M* = 2.94, *SD* = 1.07), *F*(1, 180) = 43.47, *p* < 0.001, *ŋ_p_^2^* = 0.195.

**Table 2:** Means and standard deviations of each variable.

Type	Beauty		Pleasure		Humor		Surprise		Attractiveness	
	*M*	*SD*	*M*	*SD*	*M*	*SD*	*M*	*SD*	*M*	*SD*
Beautiful	4.28	0.68	4.14	0.80	2.89	1.05	2.26	1.01	4.02	0.83
Ugly-cute	2.68	1.00	3.51	1.02	3.96	0.92	2.91	1.04	3.18	1.08
Ugly	1.80	0.90	1.80	0.83	1.99	0.87	2.94	1.07	1.79	0.87

#### 3.1.2 The mediating effect of pleasure on general beauty and attractiveness

Pearson’s correlation was utilized to calculate the correlation coefficients among various variables. The results revealed significant correlations between aesthetic appeal and cuteness, humor, pleasure, and attractiveness (see Table 3).

**Table 3:** Correlation matrix of each variable.

	1	2	3	4	5	6
1. Beauty	–					
2. Cuteness	0.443**	–				
3. Humor	0.296**	0.421**	–			
4. Surprise	−0.079	−0.127**	0.300**	–		
5. Pleasure	0.719**	0.625**	0.550**	0.006	–	
6. Attractiveness	0.636**	0.563**	0.476**	−0.003	0.733**	–

*N* = 181, **p < 0.01.

According to the bias-corrected non-parametric percentile bootstrap method recommended by Wen and Ye (2014) for testing the mediation effect, we applied the SPSS macro PROCESS with Model 6 to conduct mediation effect tests for the four variables (Hayes, 2017). The results reveal that pleasure plays a mediating role in the relationship between beauty and attractiveness, indicating that both have an impact through the experience of pleasure.

First, the independent, dependent, and mediator variables were standardized. Then, 5,000 resamples were obtained and a 95% confidence interval was calculated. The regression analysis results showed that the prediction of attractiveness by beauty was not significant [*b* = 0.11, *t*(362) = 1.52, *p* = 0.129], while pleasure [*b* = 0.88, *t*(362) = 28.60, *p* < 0.001] and humor [*b* = 0.06, *t*(362) = 2.39, *p* < 0.001] significantly predicted attractiveness. Beauty positively predicted humor [*b* = 0.90, *t*(362) = 8.87, *p* < 0.001] and pleasure [*b* = 1.91, *t*(362) = 24.25, *p* < 0.001], and humor also positively predicted pleasure [*b* = 0.47, *t*(362) = 12.65, *p* < 0.001]. Mediating effect analysis showed (Table 4) that humor and pleasure mediated the effect between beauty and attractiveness, with a mediating effect of 2.12, accounting for 94.90% of the total effect of beauty on attractiveness. Specifically, the mediating effect was mainly from this pathway: indirect effect 2 (75.65%) through the pathway of beauty → pleasure → attractiveness, whereas the mediating effect from other two pathways was although significant, the effect sizes were very small (2.69 and 16.56%): indirect effect 1 through the pathway of beauty → humor → attractiveness, and indirect effect 3 through the pathway of beauty → humor → pleasure → attractiveness.

**Table 4:** Bootstrap 95% confidence intervals for mediation effect paths.

	Effect	BootSE	BootLLCI	BootULCI	Relative mediating effect
Total	2.12	0.10	1.93	2.32	94.90%
Ind1: beauty → humor → attractiveness	0.06	0.02	0.02	0.10	2.69%
Ind2: beauty → pleasure → attractiveness	1.69	0.11	1.48	1.92	75.65%
Ind3: beauty → humor → pleasure → attractiveness	0.37	0.05	0.27	0.48	16.56%

### 3.2 Ugly-cute and attractiveness

#### 3.2.1 Differential analysis general beauty and ugly

To compare the differences between the ugly-cute and ugly groups, we utilized a repeated measure analysis of variance to assess differences in perceived beauty, emotional responses, and attractiveness under two conditions. The results showed significant differences in aesthetics, with the ugly-cute group (*M* = 2.68, *SD* = 1.00) and the ugly group (*M* = 1.80, *SD* = 0.90), *F*(1, 180) = 119.41, *p* < 0.001, *ŋ_p_^2^* = 0.399. In perceived attractiveness, the ugly-cute group (*M* = 3.18, *SD* = 1.08) differed significantly from the ugly group (*M* = 1.79, *SD* = 0.87), *F*(1, 180) = 259.36, *p* < 0.001, *ŋ_p_*^2^ = 0.590. Moreover, in pleasure, the ugly-cute group (*M* = 3.51, *SD* = 1.02) significantly differed from the ugly group (*M* = 1.80, *SD* = 0.83), *F*(1, 180) = 414.31, *p* < 0.001, *ŋ_p_*^2^ = 0.697. In humor, the ugly-cute group (*M* = 3.96, *SD* = 0.92) was significantly higher than the ugly group (*M* = 1.99, *SD* = 0.87), *F*(1, 180) = 555.82, *p* < 0.001, *ŋ_p_*^2^ = 0.755; however, there was no significant difference in surprise, *F*(1, 180) = 0.12, *p* = 0.731, *ŋ_p_*^2^ = 0.001.

Additionally, the differences between the ugly-cute and beautiful groups were compared. A repeated measure analysis of variance revealed significant differences in aesthetics, with the ugly-cute group (*M* = 2.68, *SD* = 1.00) and the beautiful group (*M* = 4.28, *SD* = 0.68), *F*(1, 180) = 482.95, *p* < 0.001, *ŋ_p_*^2^ = 0.728. In perceived attractiveness, the ugly-cute group (*M* = 3.18, *SD* = 1.08) significantly differed from the beautiful group (*M* = 4.02, *SD* = 0.83), *F*(1, 180) = 99.13, *p* < 0.001, *ŋ_p_*^2^ = 0.355. In pleasure, the ugly-cute group (*M* = 3.51, *SD* = 1.02) significantly differed from the beautiful group (*M* = 4.14, *SD* = 0.80), *F*(1, 180) = 64.87, *p* < 0.001, *ŋ_p_*^2^ = 0.265. In humor, the ugly-cute group (*M* = 3.96, *SD* = 0.92) was significantly higher than the beautiful group (*M* = 2.89, *SD* = 1.05), *F*(1, 180) = 106.62, *p* < 0.001, *ŋ_p_*^2^ = 0.372. In surprise, the ugly-cute group (*M* = 2.91, *SD* = 1.04) was significantly higher than the beautiful group (*M* = 2.26, *SD* = 1.01), *F*(1, 180) = 71.11, *p* < 0.001, *ŋ_p_*^2^ = 0.283.

#### 3.2.2 The mediating effect of humor and pleasure on ugly-cute and attractiveness

Similarly, SPSS macro-PROCESS with Model 6 was conducted to test mediation effect between ugly-cute and attractiveness. First, the independent, dependent, and mediator variables were standardized. Then, 5,000 resamples were obtained and a 95% confidence interval was calculated. The regression analysis results showed that ugly-cute significantly and positively predicted attractiveness [*b* = 0.08, *t*(362) = 2.31, *p* = 0.022] and humor [*b* = 0.98, *t*(362) = 20.89, *p* < 0.001], while its prediction for pleasure was not significant [*β* = −0.01, *t*(362) = −0.10, *p* = 0.922]. Humor significantly and positively predicted pleasure [*b* = 0.33, *t*(362) = 6.39, *p* < 0.001]. Pleasure [*b* = 0.90, *t*(362) = 31.57, *p* < 0.001] significantly and positively predicted attractiveness, while humor’s prediction for attractiveness was not significant [*b* = 0.05, *t*(362) = 1.82, *p* = 0.070].

Mediating effect analysis showed (Table 5) that humor and pleasure mediated the effect between ugly-cute and attractiveness, with a mediating effect of 0.33, accounting for 79.85% of the total effect of ugly-cute on attractiveness. Specifically, the mediating effect was from this pathway: indirect effect 3 (70.17%) through the pathway of ugly-cute → humor →pleasure → attractiveness, whereas the mediating effect from other two pathways was not significant: indirect effect 1 through the pathway of ugly-cute → humor → attractiveness, and indirect effect 2 through the pathway of ugly-cute → humor → attractiveness.

**Table 5:** Bootstrap 95% confidence intervals for mediation effect paths.

	Effect	BootSE	BootLLCI	BootULCI	Relative mediating effect
Total	0.33	0.05	0.23	0.44	79.85%
Ind1: ugly-cute → humor → attractiveness	0.05	0.03	−0.01	0.12	-
Ind2: ugly-cute → pleasure → attractiveness	−0.01	0.06	−0.12	0.11	-
Ind3: ugly-cute → humor → pleasure →attractiveness	0.29	0.05	0.19	0.40	70.17%

In addition, mediating effect analysis of surprise was conducted and the results showed that the 95% confidence interval ([−0.02, 0.02]) included 0, and this mediating effect was not significant. This finding suggests that the impact of ugly-cute on attractiveness does not occur through the experience of surprise.

Our research findings indicate that general beauty primarily elicits attractiveness through pleasure, while ugly-cute primarily generates attractiveness through the mediation of humor and pleasure rather than directly through pleasure.

## 4 Discussion

Internet memes play a significant role in today’s society as a rapidly spreading and highly influential phenomenon (Cancelas-Ouviña, 2021). Aesthetic category memes have constituted a notable shift in the recent history of social media (Schonig, 2020). They facilitate the creation of aesthetic category activities in which users engage in positive and reflective aesthetic perceptions through aesthetic experiences that differ from everyday aesthetic judgments. For instance, when we casually label a photo as “interesting” or express admiration for the “cuteness” of a puppy, these judgments often flow effortlessly from our lips, rarely prompting us to pause and contemplate what makes the puppy adorable and what implications such judgments carry (Ngai, 2012). However, with aesthetic category memes, labeling an image as “wonderfully satisfying” or “slightly amusing” entails playful and pleasurable reflections on the ambiguous criteria of these peculiar categories, as well as broader reflections on aesthetic competence. According to Schonig (2020), when appreciating aesthetic category memes, people are not only drawn to the memes themselves but also engage in thinking and pondering the standards and criteria of these peculiar categories. This process of reflection brings about a unique sense of joy, as challenging and rethinking general beauty concepts and norms enables individuals to gain a deeper understanding and experience of aesthetic competence.

In psychology and neuroscience, beauty is currently understood as a form of sensory evaluation, which is an emotional response that people may experience when encountering sensory objects (Skov and Nadal, 2021). Makin (2017) posits that the field of empirical aesthetics is overly focused on analyzing and quantifying individual parts of aesthetic objects, neglecting the indispensable emotional responses in aesthetic experiences. To the best of our knowledge, this is the first attempt to investigate the emotional mechanisms of ugly-cute memes. In this study, we compared “beautiful-uncute,” “ugly-uncute,” and “ugly-cute” memes. Here, “beautiful-uncute” refers to memes with a high aesthetic level that lacks the attribute of being cute according to general aesthetic concepts. Conversely, “ugly-uncute” refers to memes with a low aesthetic level that also lacks the attribute of being “cute.” The research findings reveal that beauty primarily generates attractiveness through feelings of pleasure, which is consistent with previous studies. Psychological theories suggest that pleasure plays a crucial role in aesthetic experiences and art appreciation (Jacobsen et al., 2004; Leder, 2013; Pelowski et al., 2016). The theory of aesthetic processing fluency explicitly equates beauty with “aesthetic pleasure”(Reber et al., 2004). Additionally, aesthetic preferences and other sensory evaluations engage the reward system, which is associated with experiences of pleasure. Functional magnetic resonance imaging studies have demonstrated that the evaluation of an object’s value, that is, the pleasure it elicits, is critical for aesthetic judgment (Brown et al., 2011). Evidence from human neuroimaging experiments suggests that aesthetic evaluation relies on the involvement of neural nuclei, resulting in varying degrees of pleasure and displeasure (Chatterjee and Vartanian, 2016). Research in psychology, neuroscience, and biology indicates that beauty should be considered a fundamental form of hedonic value shared by humans and other animals: only sensory objects that evoke pleasure are experienced as beautiful (Skov and Nadal, 2021). Similarly, research on the aesthetics of music indicates that pleasure (i.e., activity in the reward circuitry) is necessary for experiencing beauty (Mallik et al., 2017). Our research indicates that “general beauty” directly arouses attractiveness by evoking a sense of pleasure, which aligns with our first hypothesis.

In addition, our research suggests that ugly-cute memes do not directly generate attractiveness through pleasure but rather through the mediating effect of humor, which aligns with our hypothesis 2. According to Skov and Nadal (2021), experiencing an object as pleasurable is a prerequisite for judging it as beautiful. “Ugly-cute” is not conventionally beautiful, and thus it does not directly evoke attractiveness, aligning with them. However, our study indicates that ugly-cute can induce pleasure through the mediating effect of humor, subsequently stimulating attractiveness. Cela-Conde et al. (2011) propose that two distinct cognitive processes occur during aesthetic experiences, which take place at different time intervals (Cela-Conde et al., 2011): first, a general evaluation of aesthetic quality (i.e., perceiving visual stimuli as beautiful or not), referred to as “strictly aesthetic appreciation”; and then, a further evaluation of the details of the aesthetic experience (i.e., whether it is interesting or unique), known as “broadly aesthetic appreciation.” We speculate that in the aesthetic experience of ugly-cute memes, further evaluation of the amusement and uniqueness of the details may play a crucial role. Ugliness is a complex and multilayered concept, similar to beauty. The “ugliness paradox” suggests that the visual attributes of ugly objects, which are chaotic or incongruent, can cause cognitive disruption, but this cognitive disruption simultaneously stimulates people’s imagination. In simple terms, we can appreciate something that we initially dislike or even find repulsive (Felisberti, 2022). This may help explain why many ugly-cute memes appear visually unattractive or even ugly but still manage to evoke attractiveness through the mediating effect of humor. In summary, objects with general beauty typically evoke attractiveness through pleasure, whereas those aesthetically unpleasant can be transformed into the “ugly-cute” category by incorporating humor. This transformation allows them to generate attractiveness through a sequential interplay of humor and pleasure, providing valuable insights for internet meme design. Notably, ugly-cute is distinct from general beauty and mere comedy; while humorous, it uniquely triggers pleasure, offering a distinct aesthetic experience, different from humorous images popular for their direct comedic appeal.

However, this study did not find a significant role for surprise. Although there was a significant main effect of meme type on surprise ratings (beautiful group received significantly lower surprise ratings compared to ugly and ugly-cute group), there was no significant difference in surprise ratings between beautiful group and ugly-cute group. Furthermore, the results indicated that surprise did not mediate the relationship between meme type and attractiveness. This suggests that the ugly-cute phenomenon does not rely on the psychological aspect of “exceeding expectations” to create a sense of surprise, the measurement of this element can provide insights for future research.

Interestingly, the overall attractiveness of ugly-cute group is not as high as that of beautiful group. We speculate that this may be due to individual differences in aesthetic preferences. There are significant variations in aesthetic preferences among individuals, which are primarily influenced by their cultural backgrounds (Nisbett and Masuda, 2003; Bao et al., 2016). Additionally, aesthetic preferences differ according to individual personality traits (Chamorro-Premuzic et al., 2009; Myszkowski and Zenasni, 2016), gender and age (Little et al., 2015), professional knowledge (Palmer and Griscom, 2013), as well as environmental factors and personal experiences (Cooper and Maurer, 2008). The enjoyment of negative emotions in art and literature differs from the direct pleasure derived from sensory features, as it requires conscious preferences and attitudes toward the aesthetic object (Brattico and Vuust, 2017). Dietrich and Knieper (2021) argue that judgments of beauty have become increasingly subjective, with popular culture being a significant influencing factor for individualistic tendencies (Maase, 2008). Ugly-cute memes belong to popular culture and rely more on users’ cognitive perceptions, thereby exhibiting greater individual differences and resulting in a slightly lower overall level of attractiveness compared to general beauty.

Our main limitation is that we do not consider individual differences among users. Research has shown that the positive emotions generated during individual aesthetic experiences are related to curiosity (Fayn et al., 2018). Future studies can consider individual differences, including curiosity, and differentiate between individuals with high and low attractiveness to specific memes. Besides, Lastly, there could be many factors contributing to the attractiveness of ugly-cute memes that we did not test. Future research could conduct a comprehensive analysis of different variables to more fully reveal the internal mechanisms by which these memes, divergent from general beauty, evoke aesthetic attractiveness. Furthermore, aesthetics is one of the fundamental human values (Shusterman and Tomlin, 2007). However, the memes we selected were mostly morally neutral, and we did not consider the moral factors of ugly-cute memes. Research has shown that moral and aesthetic judgments are similarly influenced by negative emotions (Gollwitzer et al., 2020), and Dorado et al. (2023) further demonstrate that individuals’ sensitivity to anger and fear predicts moral judgments, while sensitivity to disgust predicts aesthetic judgments (Dorado et al., 2023). Considering that ugly-cute memes often have metaphorical implications, we speculate that the implicit moral factors and individual sensitivity associated with them may significantly influence aesthetic judgments. Future studies should consider these factors. Finally, our study predominantly focuses on a young demographic and is not applicable to the entire age spectrum. Aesthetic preferences may vary across different age groups (Aleem et al., 2020), and future research could endeavor to explore these differences throughout the entire lifespan.

## Data availability statement

The raw data supporting the conclusions of this article will be made available by the authors, without undue reservation.

## Ethics statement

The studies involving humans were approved by the Ethics Review Committee of Guangdong University of Technology. The studies were conducted in accordance with the local legislation and institutional requirements. The participants provided their written informed consent to participate in this study.

## Author contributions

JL: Conceptualization, Writing – original draft. YA: Formal analysis, Investigation, Writing – original draft. TX: Conceptualization, Formal analysis, Funding acquisition, Writing – original draft.

## References

[ref1] Akram U. Drabble J. (2022). Mental health memes: beneficial or aversive in relation to psychiatric symptoms? Human. Soc. Sci. Commun. 9:370. doi: 10.1057/s41599-022-01381-4, PMID: 36258776 PMC9559152

[ref2] Akram U. Drabble J. Cau G. Hershaw F. Rajenthran A. Lowe M.. (2020). Exploratory study on the role of emotion regulation in perceived valence, humour, and beneficial use of depressive internet memes in depression. Sci. Rep. 10:899. doi: 10.1038/s41598-020-57953-4, PMID: 31965036 PMC6972852

[ref3] Aleem H. Correa-Herran I. Grzywacz N. M. (2020). A theoretical framework for how we learn aesthetic values. Front. Hum. Neurosci. 14:345. doi: 10.3389/fnhum.2020.00345, PMID: 33061898 PMC7518219

[ref4] Armstrong T. Detweiler-Bedell B. (2008). Beauty as an emotion: the exhilarating Prospect of mastering a challenging world. Rev. Gen. Psychol. 12, 305–329. doi: 10.1037/a0012558

[ref5] Bao Y. Yang T. Lin X. Fang Y. Wang Y. Pöppel E.. (2016). Aesthetic preferences for eastern and Western traditional visual art: identity matters. Front. Psychol. 7:1596. doi: 10.3389/fpsyg.2016.01596, PMID: 27812339 PMC5071313

[ref6] Bara I. Binney R. J. Ward R. Ramsey R. (2022). A generalised semantic cognition account of aesthetic experience. Neuropsychologia 173:108288. doi: 10.1016/j.neuropsychologia.2022.108288, PMID: 35690113

[ref7] Bayley S. (2012). Ugly, the aesthetics of everything. Goodman Fiell. New York: The Overlook Press.

[ref8] Brady E. Prior J. (2020). Environmental aesthetics: a synthetic review. People Nat. 2, 254–266. doi: 10.1002/pan3.10089

[ref9] Brattico E. Vuust P. (2017). The urge to judge: why the judgmental attitude has anything to do with the aesthetic enjoyment of negative emotions. Behav. Brain Sci. 40:e353. doi: 10.1017/S0140525X17001613, PMID: 29342780

[ref10] Brielmann A. A. Nuzzo A. Pelli D. G. (2021). Beauty, the feeling. Acta Psychol. 219:103365. doi: 10.1016/j.actpsy.2021.103365, PMID: 34246875 PMC8514293

[ref11] Brown S. Gao X. Tisdelle L. Eickhoff S. B. Liotti M. (2011). Naturalizing aesthetics: brain areas for aesthetic appraisal across sensory modalities. NeuroImage 58, 250–258. doi: 10.1016/j.neuroimage.2011.06.012, PMID: 21699987 PMC8005853

[ref12] Cancelas-Ouviña L.-P. (2021). Humor in times of COVID-19 in Spain: viewing coronavirus through memes disseminated via WhatsApp. Front. Psychol. 12:611788. doi: 10.3389/fpsyg.2021.611788, PMID: 33868083 PMC8047868

[ref13] Carbon C.-C. (2011). Cognitive mechanisms for explaining dynamics of aesthetic appreciation. I-Perception 2, 708–719. doi: 10.1068/i0463aap, PMID: 23145254 PMC3485809

[ref14] Cela-Conde C. J. Agnati L. Huston J. P. Mora F. Nadal M. (2011). The neural foundations of aesthetic appreciation. Prog. Neurobiol. 94, 39–48. doi: 10.1016/j.pneurobio.2011.03.003, PMID: 21421021

[ref15] Chamorro-Premuzic T. Reimers S. Hsu A. Ahmetoglu G. (2009). Who art thou? Personality predictors of artistic preferences in a large UK sample: the importance of openness. Br. J. Psychol. 100, 501–516. doi: 10.1348/000712608X366867 19026107

[ref16] Chang C.-Y. Chan Y.-C. Chen H.-C. (2024). The differential processing of verbal jokes by neural substrates in indigenous and Han Chinese populations: an fMRI study. Behav. Brain Res. 457:114702. doi: 10.1016/j.bbr.2023.114702, PMID: 37813282

[ref17] Chatterjee A. Vartanian O. (2016). Neuroscience of aesthetics: neuroscience of aesthetics. Ann. N. Y. Acad. Sci. 1369, 172–194. doi: 10.1111/nyas.13035 27037898

[ref18] Cooper P. A. Maurer D. (2008). The influence of recent experience on perceptions of attractiveness. Perception 37, 1216–1226. doi: 10.1068/p5865 18853557

[ref19] Declos A. (2014). The aesthetic and cognitive value of surprise. Proc. Eur. Soc. Aesthetics. 6, 52–69.

[ref20] Dietrich P. Knieper T. (2021). (Neuro)aesthetics: beauty, ugliness, and ethics. PsyCh J. 11, 619–627. doi: 10.1002/pchj.478 34414671

[ref21] Dorado A. Skov M. Rosselló J. Nadal M. (2023). Defensive emotions and evaluative judgements: sensitivity to anger and fear predicts moral judgements, whereas sensitivity to disgust predicts aesthetic judgements. Br. J. Psychol. 114, 1–20. doi: 10.1111/bjop.12590, PMID: 36609781 PMC10087598

[ref22] Douglas N. (2014). It’s supposed to look like shit: the internet ugly aesthetic. J. Vis. Cult. 13, 314–339. doi: 10.1177/1470412914544516

[ref23] Eisend M. (2009). A meta-analysis of humor in advertising. J. Acad. Mark. Sci. 37, 191–203. doi: 10.1007/s11747-008-0096-y

[ref24] Fan N. (2021). Ne Zha’s image transformation in Chinese animation cinema (1961–2019). Film Fash. Consumpt. 10, 277–298. doi: 10.1386/ffc_00025_1

[ref25] Fayn K. Silvia P. J. Erbas Y. Tiliopoulos N. Kuppens P. (2018). Nuanced aesthetic emotions: emotion differentiation is related to knowledge of the arts and curiosity. Cognit. Emot. 32, 593–599. doi: 10.1080/02699931.2017.1322554, PMID: 28488919

[ref26] Felisberti F. M. (2022). Experiences of ugliness in nature and urban environments. Empir. Stud. Arts 40, 192–208. doi: 10.1177/02762374211001798

[ref27] Gollwitzer A. Marshall J. Bargh J. A. (2020). Pattern deviancy aversion predicts prejudice via a dislike of statistical minorities. J. Exp. Psychol. Gen. 149, 828–854. doi: 10.1037/xge0000682, PMID: 31580101

[ref28] Gordon M. (2012). Exploring the relationship between humor and aesthetic experience. J. Aesthet. Educ. 46, 110–121. doi: 10.5406/jaesteduc.46.1.0110

[ref29] Gordon M. (2013). Humor, laughter and human flourishing: A philosophical exploration of the laughing animal. Springer Science & Business Media.

[ref30] Graf L. K. M. Landwehr J. R. (2017). Aesthetic pleasure versus aesthetic interest: the two routes to aesthetic liking. Front. Psychol. 8:15. doi: 10.3389/fpsyg.2017.00015, PMID: 28194119 PMC5276863

[ref31] Hayes A. F. (2017). Introduction to mediation, moderation, and conditional process analysis: a regression-based approach. New York, NY: Guilford Publications.

[ref32] Hoegg J. Alba J. W. Dahl D. W. (2010). The good, the bad, and the ugly: influence of aesthetics on product feature judgments. J. Consum. Psychol. 20, 419–430. doi: 10.1016/j.jcps.2010.07.002

[ref33] Jacobsen T. Buchta K. Köhler M. Schröger E. (2004). The primacy of beauty in judging the aesthetics of objects. Psychol. Rep. 94, 1253–1260. doi: 10.2466/pr0.94.3c.1253-1260, PMID: 15362400

[ref34] Leder H. (2013). Next steps in neuroaesthetics: which processes and processing stages to study? Psychol. Aesthet. Creat. Arts 7, 27–37. doi: 10.1037/a0031585

[ref35] Leder H. Belke B. Oeberst A. Augustin D. (2004). A model of aesthetic appreciation and aesthetic judgments. Br. J. Psychol. 95, 489–508. doi: 10.1348/0007126042369811 15527534

[ref36] Leder H. Ring A. Dressler S. G. (2013). See me, feel me! Aesthetic evaluations of art portraits. Psychol. Aesthet. Creat. Arts 7, 358–369. doi: 10.1037/a0033311

[ref37] Lenda M. Skórka P. Mazur B. Sutherland W. Tryjanowski P. Moroń D.. (2020). Effects of amusing memes on concern for unappealing species. Conserv. Biol. 34, 1200–1209. doi: 10.1111/cobi.13523, PMID: 32348597

[ref38] Lieber-Milo S. Nittono H. (2019). From a word to a commercial power—a brief introduction to the kawaii aesthetic in contemporary Japan. Innovative Research in Japanese Studies, 3, 13–32.

[ref39] Little A. C. Caldwell C. A. Jones B. C. DeBruine L. M. (2015). Observer age and the social transmission of attractiveness in humans: younger women are more influenced by the choices of popular others than older women. Br. J. Psychol. 106, 397–413. doi: 10.1111/bjop.12098, PMID: 25314951

[ref40] Maase K. (2008). “The beauty of the popular. Aesthetic experience of the present age” in Die Schönheiten des Populären: Ästhetische Erfahrung der Gegenwart Frankfurt am Main: Campus-Verlag.

[ref41] Makin A. D. J. (2017). The gap between aesthetic science and aesthetic experience. J. Conscious. 24, 184–213.

[ref42] Mallik A. Chanda M. L. Levitin D. J. (2017). Anhedonia to music and mu-opioids: evidence from the administration of naltrexone. Sci. Rep. 7:41952. doi: 10.1038/srep41952, PMID: 28176798 PMC5296903

[ref43] Markovic S. (2010). Aesthetic experience and the emotional content of paintings. Psihologija 43, 47–64. doi: 10.2298/PSI1001047M

[ref44] Marković S. (2012). Components of aesthetic experience: aesthetic fascination, aesthetic appraisal, and aesthetic emotion. I-Perception 3, 1–17. doi: 10.1068/i0450aap, PMID: 23145263 PMC3485814

[ref45] Marmur O. Koichu B. (2016). Surprise and the aesthetic experience of university students: a design experiment. J. Hum. Math. 6, 127–151. doi: 10.5642/jhummath.201601.09

[ref46] Menninghaus W. Wagner V. Wassiliwizky E. Schindler I. Hanich J. Jacobsen T.. (2019). What are aesthetic emotions? Psychol. Rev. 126, 171–195. doi: 10.1037/rev0000135 30802122

[ref47] Myszkowski N. Zenasni F. (2016). Individual differences in aesthetic ability: the case for an aesthetic quotient. Front. Psychol. 7:750. doi: 10.3389/fpsyg.2016.00750, PMID: 27242647 PMC4871874

[ref48] Ngai S. (2012). Our aesthetic categories: zany, cute, interesting. Cambridge. Mass.: Harvard.

[ref49] Nisbett R. E. Masuda T. (2003). Culture and point of view. Proc. Natl. Acad. Sci. 100, 11163–11170. doi: 10.1073/pnas.1934527100, PMID: 12960375 PMC196945

[ref50] Nittono H. Lieber-Milo S. Dale J. P. (2021). Cross-cultural comparisons of the cute and related concepts in Japan, the United States, and Israel. SAGE Open 11:215824402098873. doi: 10.1177/2158244020988730

[ref51] Nittono H. Saito H. Ihara N. Fenocchio D. N. Andreau J. M. (2023). English and Spanish adjectives that describe the Japanese concept of kawaii. SAGE Open 13:215824402311524. doi: 10.1177/21582440231152415

[ref52] Palmer S. E. Griscom W. S. (2013). Accounting for taste: individual differences in preference for harmony. Psychon. Bull. Rev. 20, 453–461. doi: 10.3758/s13423-012-0355-2, PMID: 23242798

[ref53] Pelowski M. Markey P. S. Lauring J. O. Leder H. (2016). Visualizing the impact of art: an update and comparison of current psychological models of art experience. Front. Hum. Neurosci. 10:160. doi: 10.3389/fnhum.2016.00160, PMID: 27199697 PMC4844603

[ref54] Perlovsky L. (2014). Mystery in experimental psychology, how to measure aesthetic emotions? Front. Psychol. 5:1006. doi: 10.3389/fpsyg.2014.01006, PMID: 25309479 PMC4159989

[ref55] Reber R. Schwarz N. Winkielman P. (2004). Processing fluency and aesthetic pleasure: is beauty in the Perceiver’s processing experience? Personal. Soc. Psychol. Rev. 8, 364–382. doi: 10.1207/s15327957pspr0804_3, PMID: 15582859

[ref56] Sabina G. (2019). Deep in mummy matters. UGLYDOLLS giveaway and activity sheets. Available at: https://deepinmummymatters.com/uglydolls-giveaway-and-activity-sheets/

[ref57] Schindler I. Hosoya G. Menninghaus W. Beermann U. Wagner V. Eid M.. (2017). Measuring aesthetic emotions: a review of the literature and a new assessment tool. PLoS One 12:e0178899. doi: 10.1371/journal.pone.0178899, PMID: 28582467 PMC5459466

[ref58] Schonig J. (2020). “Liking” as creating: on aesthetic category memes. New Media Soc. 22, 26–48. doi: 10.1177/1461444819855727

[ref59] Shifman L. (2013). Memes in a digital world: reconciling with a conceptual troublemaker. J. Comput.-Mediat. Commun. 18, 362–377. doi: 10.1111/jcc4.12013

[ref60] Shusterman R. Tomlin A. (Eds.) (2007). Aesthetic experience. 1st Edn New York: Routledge.

[ref61] Silvia P. J. Fayn K. Nusbaum E. C. Beaty R. E. (2015). Openness to experience and awe in response to nature and music: personality and profound aesthetic experiences. Psychol. Aesthet. Creat. Arts 9, 376–384. doi: 10.1037/aca0000028

[ref62] Skov M. Nadal M. (2021). The nature of beauty: behavior, cognition, and neurobiology. Ann. N. Y. Acad. Sci. 1488, 44–55. doi: 10.1111/nyas.14524, PMID: 33147651

[ref63] Suls J. M. (1972). “A two-stage model for the appreciation of jokes and cartoons: an information-processing analysis” in The psychology of humor (Elsevier), Theoretical perspectives and empirical issues, 1, 81–100.

[ref64] Tiihonen M. Haumann N. T. Shtyrov Y. Vuust P. Jacobsen T. Brattico E. (2024). The impact of crossmodal predictions on the neural processing of aesthetic stimuli. Philos. Trans. R. Soc. Lond. Ser. B Biol. Sci. 379:20220418. doi: 10.1098/rstb.2022.0418, PMID: 38104610 PMC10725772

[ref65] Wen Z. Ye B. (2014). Analyses of mediating effects: the development of methods and models. Adv. Psychol. Sci. 22:731. doi: 10.3724/SP.J.1042.2014.00731

